# The virtues and limits of specialization in global value chains: analysis and policy implications

**DOI:** 10.1007/s40812-022-00247-9

**Published:** 2023-01-03

**Authors:** Andrea Coveri, Antonello Zanfei

**Affiliations:** Department of Economics, Society, Politics, University of Urbino “Carlo Bo”, Urbino, Italy

**Keywords:** Specialization, Diversification, Global value chains, Functions, Innovation, Resilience, F12, F60, O14

## Abstract

A growing concern has emerged in both academic research and policy circles about the hidden risks that can arise from a narrow specialization of economies in a world characterized by the international fragmentation of production. In this work, we address the virtues and limits of specialization in light of the strong interdependencies between countries induced by the emergence and evolution of global value chains (GVCs). The need to shift the focus from the product level to the functional level is discussed from both a conceptual and empirical perspective. Moreover, several arguments are advanced in favour of functional diversification. It is argued that economies performing a relatively large range of value adding activities are in a better position to foster process and product innovation and increase the resilience of the productive structure in face of both domestic and external shocks. Accordingly, we provide a stronger conceptual basis for industrial policies aimed to address the vulnerability of GVCs in times of major disruptive events. We stress that responding to these shocks implies a careful definition of the geographical boundaries of international production networks, substantial investments in strategic activities at the national and macro-regional levels, as well as a more selective sourcing of inputs and knowledge assets on a global scale.

## Introduction

Since the mid-1980s, the increasing international fragmentation of production and the advent of global value chains (GVCs) (Feenstra, 1998; Timmer et al., 2014) have induced a growing specialization of economies at the level of value adding activities, also called “functions” or “tasks” (Grossman & Rossi-Hansberg, 2006; Sturgeon, 2008; Sturgeon & Gereffi, 2009; Timmer et al., 2019; Coveri & Zanfei, 2022b). This has determined *inter alia* a greater inter-dependence across world economies for the procurement of critical production inputs, advanced materials, components, as well as know-how (OECD, 2013; UNCTAD, 2013).

However, a growing concern has emerged in both academic research and policy circles about the fragility of GVCs in times of global crises. The vulnerability of global production networks had already emerged as a result of tensions at the turn of the XXI century, which has reached a climax with the great recession that erupted in 2008. Recent disruptive events have further highlighted this fragility, particularly in the case of the outbreak of Covid-19 pandemic (Coveri et al., 2020; Giammetti et al., 2020; Strange, 2020; Gereffi et al., 2022) and the war triggered by the Russian invasion of Ukraine (Mariotti, 2022; Sturm, 2022; Tajoli, 2022).

Policy initiatives have proliferated with the aim of increasing control over strategic GVC activities at the national and macro-regional levels in order to improve the capacity of economies to respond to unexpected events and global challenges. In the USA, the White House released in June 2021 a report entitled “Building Resilient Supply Chains, Revitalizing American Manufacturing, and Fostering Broad-Based Growth”.^1^ The report addresses the supply chain fragilities in four key industries, i.e., semiconductor manufacturing and advanced packaging, batteries for electric vehicles, critical mineral and materials used in high-tech ICT products and active pharmaceutical ingredients. Policy recommendations have been made with the explicit objective of rebuilding and strengthening the resilience of the US industrial base and innovation capabilities in these key productions. The European Union has also acted in a similar direction. Since 2019, the European Commission has detected six “Key Strategic Value Chains” – i.e., autonomous vehicles, hydrogen technologies and systems, health, Internet of Things (IoT), low-CO2 emission industry, and cybersecurity – whose strength and resilience are considered crucial for the “strategic autonomy” of the EU. Notably, the Strategic Forum for Important Projects of Common European Interest released in November 2019 a report entitled “Strengthening Strategic Value Chains for a future-ready EU Industry”,^2^ according to which “strategic autonomy requires avoiding critical industrial and technological dependence from third countries” (p. 15), specifying also that this policy project concerns “the whole ecosystem of strategic value chains, covering the whole spectrum from research and development to manufacturing and related services” (ibid.).

These policy initiatives imply a shift away from the standard emphasis on the gains from trade that economies derive from specialization in production stages wherein they exhibit a comparative advantage. As opposed to this view, there seems to be a growing attention to the need for economies to control a wider set of strategic activities along GVCs at national and supranational levels.

In this paper, we attempt to provide a stronger conceptual basis for policies aimed to deal with the fragility of GVCs by discussing the pros and cons of specialization in a world characterized by international fragmentation of production. We shall argue that in such a world the specialization that matters concerns value adding functions more than products and industries. In addition, and most importantly, we highlight the hidden risks of *hyper-specialization* that could arise from a narrow specialization of economies in a few GVC functions among those required to bring a product or service to market.

More precisely, we advance the hypothesis that countries which develop and retain the capacity to perform a relatively large range of value adding activities are likely to be in a better position to pursue at least two key objectives: (i) fostering the pace of process and product innovation through the interaction between different functions along the value chain; (ii) increasing the resilience of the productive structure in face of both domestic and external shocks by favouring the deployment and recombination of larger sets of competencies and abilities needed to promptly adapt to changed conditions.

The arguments presented in this paper also connects to the broader debate on the future of GVCs and on the geographical extension of production networks. Even before the outbreak of the pandemic, a discussion was growing in Western countries on the opportunity to promote production reshoring with the aim of strengthening domestic innovation capabilities and reducing the strong reliance on China and other South-East Asian economies for the supply of a large array of components (see, e.g., Panwar 2010; Shih, 2014). The uncertainty induced by the advent of the Covid-19 pandemic and the geopolitical tensions due to the ongoing Russia-Ukraine war have further fueled the debate on whether to increase the resilience of GVCs by fostering the regionalization of value chain activities through friend-shoring and the near-sourcing of critical supplies for strategic industries (see, e.g., Miroudot, 2020; Shih, 2020). As will be argued, responding to these shocks implies a careful definition of the geographical boundaries of international production networks, substantial investments in strategic activities at the national and macro-regional levels, as well as selective global linkages to connect to key assets and knowledge sources whenever necessary.

The remainder of this work is organized as follows. Section 2 will briefly recall the virtues and limitations of specialisation in both standard theory and more recent approaches to trade and development. Section 3 discusses how specialisation as well as diversification arguments should be reconsidered in the light of the emergence and expansion of GVCs. Sections 4 and 5 address the implications of hyper-specialisation – arguing instead in favour of some degree of functional diversification – with specific reference to innovation capacities and resilience. Section 6 concludes.

## Specialization *versus* diversification

International trade theory has its foundations in the classical Ricardian argument that countries specialize in industries in which they exhibit a comparative (cost) advantage, namely a relatively lower opportunity cost in the production of given goods compared to other economies. This view has been incorporated in standard trade models and has long been identified with a straightforward argument in favor of specialization as the key engine of gains from trade. However, one needs to acknowledge a set of issues that are inherent to the specialization story.

First, as pointed out by the pioneers of economic development – such as Rosenstein-Rodan (1943), Prebisch (1950), Singer (1950), Myrdal (1957), Hirschman (1958) and Kaldor (1967) – not all sectors share the same growth potential. Countries that manage to specialize in industries featured by larger learning effects and higher returns to scale are better placed to foster a sustained economic development path (Amsden, 1989; Dosi, Pavitt & Soete, 1990). In a nutshell, producing ‘microchips’ is not the same as producing ‘potato chips’: as has recently been shown by Dosi, Riccio and Virgillito (2021), the quality of specialization matters, especially for the long-term development prospects of economies.

Second, gains from specialization differ according to how aggregated the analysis is. As clearly stated by Hausmann (2013), higher specialization of economic agents at the micro level goes hand in hand with larger economic diversification at the macro level. In fact, while the specialization of individual workers yields higher efficiency, this may not be the case at the firm level (depending on the nature and variety of skills they possess), and even less so at higher levels of aggregation (e.g., city, region and country scale).

In other words, the economic advantages of specialization are not as straightforward as they might appear at first glance. On the one hand, some specialization patterns are more conducive to economic development than others. On the other hand, gains from specialization of individuals co-exist with the benefits arising from diversification at more aggregated levels of analysis. These benefits stem from the broader range of activities undertaken by specialized individuals and their more numerous and complex combinations.

These two key observations lie at the basis of an expanding stream of empirical literature adopting an “economic complexity” approach to economic development and growth trajectories of countries and regions (Hausmann et al., 2007; Hidalgo et al., 2007; Hidalgo & Hausmann, 2009). The core idea is that countries equipped with a broader set of capabilities produce a larger number of products, hence they will show a more diversified export basket; by the same token, products which require more capabilities to be produced will be realized and exported by few countries, implying that these products are less ubiquitous.^3^

This line of research leads to a result quite opposite to the one reached in standard economic literature, according to which countries should specialize in the few industries in which they have a comparative advantage to improve their growth performance. In fact, the key conceptual message to be drawn from this “complexity approach” is rather that economic development of countries is driven by their level of productive diversification, especially into products that are incrementally more complex. In other terms, economic development opportunities of countries are closely linked to the amount of knowledge and productive capabilities they are equipped with, where the latter can be measured by the diversity and ubiquity of products they export.

## From product to functional diversification

Although the conceptual framework offered by the complexity approach to economic development is quite convincing, it suffers from an important shortcoming. In fact, it fails to capture a key feature of the modern real-world economy that relates to the changes in the organization of production induced by the rise of GVCs.^4^ As a growing body of literature has shown, the advent of the ICT revolution in the last decades of the XX century – together with other major geopolitical and policy-related events – has led countries to increasingly specialize in specific value adding functions, or “tasks”, involved in the GVC of products, be they a Barbie doll, a Boeing aircraft or an iPhone (Tempest, 1996; Grossman & Rossi-Hansberg, 2006; Sturgeon, 2008; Sturgeon & Gereffi, 2009; Dedrick et al., 2010; Sturgeon et al., 2013). GVC functions include all value chain stages – from research, design and development activities, to manufacturing and assembly operations, up to commercialization services – required to make and bring a product to market. For example, Timmer et al. (2019) has shown that China’s primacy in the export of electronic products is actually due to its specialization in performing the fabrication stages (mainly manufacturing and assembly) of the GVC of these goods.

Cross-border trade and capital flows are largely affected by the strategic offshoring decisions of multinational corporations (MNCs) committed to specialize in their core competencies while moving other value adding activities to other locations where the latter can be carried out at a lower cost or more effectively (Buckley et al., 2020; Coveri & Zanfei, 2022a). Symmetrically, low- and middle-income countries have been provided with the opportunity to promote exports and foster their industrialization pace by joining international supply chains without the imperative to build-up new industries from scratch (Cattaneo et al., 2010; Taglioni & Winkler, 2016).

The emergence and evolution of GVCs induces a re-consideration of the specialization *versus* diversification argument. In fact, it appears that the issue at stake is not the degree of specialization – or diversification – of economies at the product level. It is rather a matter of specializing – or diversifying – into *functions* within individual industries. This change in perspective can be at least partially reconciled with standard trade models: economies can be assumed to specialize according to their comparative advantage in selected GVC tasks rather than in entire industries or product lines (Grossman & Rossi-Hansberg, 2008; Baldwin & Evenett, 2015; Timmer et al., 2019). Consistently, by allowing each country to specialize only in those tasks in which it shows relatively higher productivity, this “unbundling” of industries into functions can be expected to lead to an amplification of the standard efficiency gains from specialization according to comparative advantages, as compared to a world of marked by specialisation at the level of entire industries or products (Baldwin, 2016).

This way of disarticulating production processes and theorizing specialization at the sub-sectoral level has its merits and convenience, as it allows to adapt standard concepts and models (also in the neoclassical domain) to a new and more complex reality. That is, it permits to use established analytical categories to a world that is less and less characterized by exchanges of wine for clothes, and more and more by transactions of parts, production stages and value adding activities (Grossman & Rossi-Hansberg, 2006). Of course, one cannot go too far adapting standard models which can hardly accommodate the heterogeneity of GVC structures, the variety of functions involved, and their distinctive role across and within industries. Identifying comparative advantages in knowledge-intensive functions is particularly tough an effort, given the well-known difficulties in assigning an economic value to knowledge assets.

In a similar vein, one might venture saying that also the economic complexity framework could be adapted to deal with the international fragmentation of production to the extent that the analysis is brought to a more disaggregated level. Individual products could be disentangled into components, so that the diversification and ubiquity of exported goods could be analyzed at a finer and finer level of disaggregation (see, e.g., Giovannetti & Vannelli 2020). This might be consistent with the idea put forth by the economic complexity literature of connecting the variety of products with distinct competencies. The analysis of underlying knowledge assets might be scaled down to account for narrower and more specific competencies that are associated to the production of components of a given good or service. For instance, one may well analyze the specialization of a country in aircraft industry by focusing on that country’s exports of all the relevant components and services that lead the production of aircrafts, and by exploring the competencies associated to each of these components and services.

Nevertheless, it appears to us that the transition from products to functions is not an easy ride and raises at least two key issues.

First, *the disaggregation of products does not properly capture GVC participation and value appropriation*. By de-composing the exports of a given country (region or firm) into finer and finer product categories one may provide a more detailed picture of specialisation of that country (region or firm) in different segments of a sector or product line. Hence, one may more precisely describe the role played by that country (region or firm) in a highly fragmented production process. However, by disaggregating product categories, one can derive no relevant information on what activities or value adding functions are being undertaken by countries (regions or firms) to bring those products to market. By disregarding the functional level of analysis, one is likely to miss a fundamental feature of the international fragmentation of production, i.e., the different economic value that is associated with distinct GVC activities.

Indeed, a key merit of the literature on GVCs has been to shed light on the links between such activities and the growth and development prospects of economies. In fact, it has pointed out that upstream functions such as R&D and product development, as well as downstream functions like commercialization and after sale activities, are associated to higher value capture opportunities than fabrication and assembly operations. Hence, countries (regions and firms) specializing in upstream and downstream activities within industries are likely to be better positioned to seize the economic value generated along GVCs and will therefore benefit from greater growth opportunities (Mudambi, 2008; Shin et al., 2012; Durand & Milberg 2020; Coveri & Zanfei, 2022b).

Second, *there are important empirical issues to be tackled when bringing the analysis from products to functions.* Besides the approaches based on the analysis of case studies concerning the GVC of a specific product (see, e.g., Dedrick et al., 2010; Ali-Yrkkö & Rouvinen, 2015), different streams of empirical literature have addressed these issues. A first stream of literature exploited international input-output tables to trace the origin of value added in export flows. In particular, the seminal contribution by Hummels, Ishii and Yi (2001) – who first introduced a measure of ‘vertical specialization in trade’ capturing the import content of exports – has paved the way for a growing literature on the measurement of ‘trade in value added’ (i.e., the value-added content of trade flows). Contributions in this field have developed new measures aimed at detecting the (geographical and sectoral) sources of (direct and indirect) value added that is embodied in gross exports and absorbed by (domestic and foreign) final demand (Johnson & Noguera, 2012; Koopman et al., 2014; Timmer et al., 2014). However, while informative of industries’ value-added contribution to exports, these indicators fail to capture the functional sources of value added, having sectors as the main unit of analysis. A second stream of contributions sought to analyze the positioning of countries (firms) in GVCs based on the degree of “upstreamness” of the industries in which the economies specialize (which the firms belong to) (Antràs et al., 2012; Rungi & Del Prete, 2018; Meng et al., 2020). Although these measures shed some light on the position of countries (firms) in GVCs, they fail to reflect what value adding activities are being carried out in a given sector of the economy under observation. In other words, one country (region of firm) may well be active and even specialized in the export of a given set of goods that can be classified “upstream” in the value chain. However, no relevant information can be provided on the tasks undertaken in the production process, hence allowing little or no inference on its functional specialization and related value capture opportunities (de Vries et al., 2021).

Recent empirical research has more directly addressed the functional profile of economies by focusing on the geography of value adding activities and on the specialization of countries and regions in such activities. Two main strands of contributions are emerging in this respect. On the one hand, Timmer et al. (2019) provided a methodology to compute the ‘functional specialization in trade’ of economies and subnational regions based on the value added embodied in exports that is generated by (or accruing to) workers belonging to different occupational categories. By so doing, the authors are able to measure the amount of value added that can be traced back to workers performing specific tasks along the GVC of exported goods and services. On the other hand, using data on foreign direct investments (FDIs) occurring in different value chain activities (ranging from R&D to manufacturing operations, up to commercialization activities), Stöllinger (2019, 2021) and Zanfei et al., (2019) have examined the geographic distribution of functions within “captive” GVCs controlled by large MNCs. These authors introduced measures of ‘functional specialization in FDI’ by adopting a cross-sectional perspective at the country-industry level (Stöllinger, 2021) and a longitudinal perspective at both country and subnational region level (Coveri & Zanfei, 2022b; Coveri et al., 2022). Noteworthy, relying on these FDI-based indicators, Grieveson et al. (2021) provided evidence that the functional specialization of the economies does not differ much across industries. Coveri and Zanfei (2022b) empirically demonstrated that greater specialization in both R&D and commercialisation functions is associated with higher value capture opportunities in GVCs – thus providing the first systematic test of the ‘smile curve’ hypothesis (Mudambi, 2008) on a global scale.

Summing up, functional specialization patterns are likely to better account for the international fragmentation of production that has occurred over the last forty years. Several contributions reviewed above have stressed the importance of switching away from products to functions and have highlighted that specializing in the most knowledge-intensive functions yields better economic outcomes and growth opportunities than specialization in more tangible-intensive activities such as fabrication operations.

But how far should a country (region or firm) move in the direction of functional specialization? Quite similar to the discussion we sketched above on the virtues and limits of (product) specialization *versus* diversification, one needs to evaluate the most appropriate *degree* of functional specialization. On the one hand, it is quite self-evident that specializing “only” in low value capturing activities has negative implications in terms of economic growth and development. This might hold also in the case of countries (regions and firms) specializing in highly dynamic industries and products: an economy that only does assembly activities in no matters how many industries is not likely to capture large shares of value even if it is a major exporter of highly sophisticated goods. On the other hand, specializing “only” in a limited range of high value adding activities (such as R&D and design functions) without any presence in lower end activities (such as manufacturing or assembly) may not allow growth opportunities to be fully exploited. In fact, there are interdependencies across functions that need to be exploited to generate valuable knowledge and capabilities. Moreover, there are many good reasons to argue that greater functional diversification could be associated with a higher degree of production flexibility and a greater capacity of the economy to respond to unexpected changes and shocks which might undermine the structure and stability of GVCs (Shih, 2020).

In what follows, we review arguments according to which the ability of countries to maintain and expand their capabilities to carry out diverse and more complex tasks represents a key factor to improve their innovation potential and resilience.

## Functional diversification and innovation

In recent years, an increasing number of studies have highlighted the risks that international production outsourcing may entail for countries’ capacity to generate innovation. Well-known contributions in this field have been provided by Pisano & Shih (2009, 2012). These authors especially focused on the “hidden damages” due to the manufacturing offshoring strategies pursued for decades by US-based MNCs. These strategies were mainly dictated by the imperative for US companies to specialize in their core competencies, such as R&D, design, as well as marketing and distribution activities, while outsourcing low value adding functions – such as manufacturing activities concerning the production of components and assembly operations – to low labour-cost countries, especially Asian economies.

According to Pisano & Shih (2012), such a strategy of dismissing manufacturing to concentrate into higher value adding activities may be rational at the micro level, i.e., if one adopts the point of view of the individual firm: the gains from reduced production costs may well outweigh the higher costs of communication, logistics, and coordination due to the offshoring of production. However, such a strategy can be destructive at the macro level, i.e., for the economic system as a whole. In fact, it contributes to the steady erosion of what they call “industrial commons”, which consist of “webs of technological know-how, operational capabilities, and specialized skills that are embedded in the workforce, competitors, suppliers, customers, cooperative R&D ventures, and universities and often support multiple industrial sectors” (Pisano & Shih, 2012, p. 13).

More specifically, they show that since the Nineties the surge in production offshoring by US companies has contributed to lower the strength of local backward and forward linkages with providers of upstream and downstream activities, up to undermine the industrial commons – namely the knowledge and productive capabilities a country is endowed with – on which the introduction of new high-tech products and processes ultimately rely upon. In this respect, Pisano & Shih (2009, 2012) provide several examples of products the US has lost or is losing the ability to manufacture, and later also to design, due to the loss of complementary assets like specialized suppliers, critical know-how and an appropriate set of workers (e.g., Windows notebook PCs). Consistently, they document that the international outsourcing of the lowest value adding functions like simple assembly has often been followed by the offshoring of higher value adding design and engineering activities related to the manufacturing process of advanced materials and components, which in turn are crucial for the development of new cutting-edge product and process innovation (Pisano & Shih, 2009).

Moreover, the seemingly rational companies’ rush towards specializing in an increasingly narrow set of core functions due to cost-reduction objectives has also hampered to a certain extent their capabilities to exploit external economies of agglomeration, i.e., the positive externalities deriving from the spatial proximity of firms, suppliers and service providers in geographically bounded places (Feldman & Kogler, 2010). This is especially true in industries where the innovation process largely relies on knowledge flows which are highly tacit, making the geographical proximity between lead firms and supply chain suppliers even more important to effectively transmit and exchange information and know-how (Dankbaar, 2007; Castellani & Lavoratori, 2020). Examples include innovation in key technologies and sectors, like the wind and solar power technology, batteries, but also in semiconductors, chemical and pharmaceutical industry, in which a close interaction of R&D and design labs with manufacturing and assembly operations, as well as with large user companies, are crucial to develop new process and product innovation (Zirpoli & Becker, 2011; Breznitz & Cowhey, 2012; Berger, 2013; Fuchs, 2014; Fagerberg, 2022).

In line with these arguments, a number of empirical studies have recently shown that the co-location of R&D and manufacturing functions, across and within industries, persists both at the national (subnational) level and across national borders (Ketokivi & Ali-Yrkko, 2009; Fuchs & Kirchain, 2010; Buciuni et al., 2013; Bailey & De Propris, 2014; Gray et al., 2015; Alcácer & Delgado, 2016; Buciuni & Finotto, 2016; Buciuni & Pisano, 2018; Delgado, 2020). The important role of co-location and interactions across different value adding activities is by and large consistent with a chain-link view of innovation, wherein continuous information exchanges and feedbacks occur along the entire process of conception, design, manufacturing and commercialization of new processes and products (Kline & Rosenberg, 1986).

Although the dynamics of innovation are largely technology- and industry-specific (Castellani et al., 2015; Delgado, 2020), as well as dependent on the structure and governance of GVCs in which firms are involved in (Ambos et al., 2021; Buciuni & Pisano, 2021), the lesson to be drawn from this literature is that the economy-wide specialization in a circumscribed set of activities can result into a narrower production matrix, reduced learning opportunities and a lower amount of tacit knowledge circulating across domestic actors. This entails the risk of hindering the capability of economies to generate innovation in the long run and should lead to a more careful assessment of the expected gains from the GVC-induced hyper-specialization.

## Functional diversification and resilience

Resilience has been defined as the ability of places to withstand economic shocks and promote rapid recovery by promptly adapting to the changed conditions (Christopherson et al., 2010; Martin, 2012). The resilience capacity, in other terms, concerns the ability of economies to maintain and recombine their existent capabilities in order to develop new growth paths after the occurrence of a disruptive event (Boschma, 2015; Xiao et al., 2018; Martin & Sunley, 2020).

Since the seminal work by Conroy (1975), an expanding literature – especially in the field of evolutionary economic geography (EEG) – has shown that the mix of economic activities and the interdependencies among them crucially affect the capacity of countries and regions to cope with adverse economic fluctuations (Dissart, 2003; Neffke et al., 2011; Kogler et al., 2017). In particular, it has been shown that a more diversified economic structure is usually better equipped than a more specialized one in terms of adaptation and recovery after a shock, especially whether the diversification occurs in “related” industries and technologies which share stronger relationships among them (e.g., Frenken et al., 2007; Balland et al., 2015; Brown & Greenbaum, 2017; Crescenzi et al., 2016; Rocchetta & Mina, 2019). This line of argument has implications for the analysis of the degree of functional diversification as well.

First, a more diversified productive structure allows economies to better diversify the risk. This is because the development of a larger number of partially uncorrelated economic activities alleviates the negative consequences which can derive from sector-specific shocks or other kinds of disruptive events that might affect selected productive technologies or occupational categories (Grabher & Stark, 1997; Boschma, 2015; Doran & Fingleton, 2018). Second, and most importantly, the countries’ capacity to sustain a higher economic diversification would reflect the availability of a larger mix of technological and organizational capabilities, which in turn can be more easily and flexibly recombined to soften the consequences of adverse phenomena and to adapt to changing economic conditions (Neffke et al., 2011; Castaldi et al., 2015). Overall, a more diversified economic structure provides greater capacity to better absorb more severe shocks in the short term and larger “adaptability” in the long run by increasing the opportunities to develop new growth paths (Pendall et al., 2010; Pike et al., 2010; Martin, 2012; Tóth et al., 2022).

Even in this context, however, the value adding functions that, within industrial sectors, are performed by companies located in a particular country or region are broadly overlooked. This means that economies showing an apparently large diversification in the economic structure might be featured by a narrow set of productive and knowledge capabilities whether they are hyper-specialized in few selected value chain functions of products. In these cases, the resilience capacity of places is likely to be overestimated.

On the one side, the nature and variety of GVC functions that countries are able to perform is likely to provide a better assessment of the domestic actors’ capabilities to absorb an adverse shock and to mobilize resources – in terms of assets, skills and knowledge – in order to more rapidly recover after an extreme event. In fact, the capabilities to carry out as diverse value chain functions as R&D activities, design, manufacturing and assembly operations, logistics and supporting services are very different and do not entail the same opportunities in terms of learning effects, returns to scale and competencies (Gereffi, 2014; Timmer et al., 2019; Stöllinger, 2021; Coveri & Zanfei, 2022b; Coveri et al., 2022).

On the other side, a greater mix of value adding functions can be expected to imply a larger capacity to promptly react to external shocks. In fact, the availability of knowledge and productive capabilities, as well as the capacity to expand and coordinate the interactions between different value adding functions, provide larger opportunities to convert domestic production in case of economic shocks, like global supply chain disruptions, artificial disasters or climate-induced extreme events (Paglialunga et al., 2022). In other terms, a greater functional diversification of economies, namely their human, physical and institutional capability to perform a larger range of value adding activities, increases the possibilities to recombine existent competencies and is therefore likely to reduce the vulnerability to adverse, unexpected phenomena (Shih, 2020).

On the contrary, the hyper-specialization in few value adding functions carries the risk of shrinking the productive matrix of economies while increasing the interdependence of countries, making them more vulnerable to external shocks and changes in global conditions (Coveri et al., 2020; Espitia et al., 2022; Borin et al., 2021).

## Conclusion

Technology- and policy-enabled unbundling of the production process have led firms to benefit from the internationally outsourcing of value chain functions which fall outside their core competencies. At the country level, this has been largely considered to be beneficial as well, since the more granular international division of labour prompted by the international fragmentation of production has allowed economies to specialize in the value adding activities in which they show a comparative advantage.

This view has been partially questioned by a growing stream of literature adopting a “complexity approach” to economic development, which has shown that the level of economic development of a country is positively associated with the diverse and non-ubiquitous nature of products it exports, which in turn exponentially increases with the amount of productive capabilities a country is equipped with (Hidalgo & Hausmann, 2009). Since economic development appears to be positively related to the diversification of the export basket of countries, less advanced economies should aim at positioning themselves in the highest possible number of GVCs (Hausmann, 2013). In this way, they could become a node in a growing number of production networks and expand their own production capabilities, as reflected by a broader export basket.

In this work, we have argued that expanding export baskets may not *per se* ensure higher growth opportunities, as these depend to a large extent on the value adding activities performed within industries, whatever the sectoral disaggregation of the analysis. In fact, a substantial expansion of the product composition of an economy’s export basket may not be associated with higher growth prospects if combined with a specialization in the least value adding activities of GVCs. Accordingly, we have stressed that, next to the diversification in terms of industry products, greater attention should be placed on the functional diversification of economies, namely the ability of countries to carry out a wider range of value adding functions.

Most importantly, we advanced the hypothesis that – while hyper-specialization of economies risks hampering their innovation rate and reducing resilience to adverse shocks – countries showing a greater diversification in terms of value chain activities might be better positioned with reference to both these dimensions. In fact, some degree of functional diversification can be associated with a broader and increasingly diverse set of skills, favours a better exploitation of backward and forward linkages between GVC stages and fosters greater learning opportunities due to the complementarities and structural interdependencies among GVC activities (Zirpoli & Becker, 2011; Breznitz & Cowhey, 2012; Pisano & Shih, 2012; Berger, 2013; Fuchs, 2014).

Moreover, focusing on value chain activities rather than end products allows to better frame the previously mentioned policy initiatives undertaken by the US and the EU to strengthen the innovation capacity and economic resilience in key targeted industries. On the one side, the emphasis placed by these Governments’ initiatives on the need to expand the value chain functions that can be carried out by local actors is in line with the perspective discussed in this work. In fact, the rationale of these policies is to increase the benefits that can derive from a larger functional diversification of economies. On the other side, a greater functional diversification of economies could entail a re-design of value chains, whose extension might be substantially reduced. In other words, economies displaying a broader functional diversification may need fewer and less geographically dispersed foreign suppliers of both tangible and intangible assets.

However, the crucial challenge for building shorter and more resilient value chains consists in the ability of economies to develop, and gain access to, the strategic resources – e.g., pools of highly skilled workers and shared infrastructures – underpinning a robust ecosystem of innovation. Hence, reducing the extension of value chains to include fewer, more reliable players on a (macro-)regional rather than a global scale implies massive investments to fill key competence gaps value chains. As a result, the possible trade-off between resilience and efficiency that might emerge in the short term – e.g., due to higher costs for firms caused by the reorganisation of industries and the time it takes to identify new reliable suppliers – is likely to be less binding in the medium to long term, as the reduced exposure to GVC disruptions due to external shocks might prevent serious collapses in firm productivity (Gölgeci et al., 2020; Ryan et al., 2022). Furthermore, such a large-scale investment strategy should go hand in hand with a more selective sourcing of raw materials, inputs and knowledge assets on a global scale (Coveri et al., 2020).

From an operational point of view and taking a cue from US and EU policy initiatives, one can envisage three main lines of government action to balance the need for resilience with the actual availability of strategic assets. First, a careful mapping of the “gaps” in value chains is needed at the macro-regional level is required in order to identify the value adding functions that domestic actors are currently unable to perform, either because those functions have been most outsourced to foreign producers or because of a strong dependence on the foreign supply of critical inputs. This phase is crucial to detect the activities that need to be stimulated to increase the pace of innovation and reduce the vulnerability and exposure of economies to abrupt changes in the global production landscape. Second, a coordinated investment plan should be promoted to expand the productive capacity of economies across value chain functions and to fill the missing capabilities in macro-regional production networks, especially in strategic industries, together with the definition of clear objectives to be achieved over different time horizons. In the case of the EU, this should be outlined in detail at supranational scale and then undertaken at both national and supranational levels to ensure a more effective implementation of coordinated and integrated efforts. Third, policies must be designed to ensure selective sourcing of key production inputs and knowledge assets on a global scale, in order to obtain and enlarge access to strategic resources that cannot be supplied by domestic producers in an effective or timely manner.

In view of this, the collection of data and the development of indicators to systematically identify missing links in value chains are essential and should be the subject of future research. On the one side, new measures that take into account the functions that economies actually perform along the GVCs of exported products would make it possible to capture the diversification of countries and regions at the industry-function level together with the “quality” of their diversification. On the other side, they would be a key tool for a better design and implementation of industrial policies aimed at strengthening the innovation capacity and resilience of economies in the face of global challenges.

## Footnotes

1 The White House report is available here: https://www.whitehouse.gov/wp-content/uploads/2021/06/100-day-supply-chain-review-report.pdf (last access: 22 October 2022).

2 The report released by the Strategic Forum for Important Projects of Common European Interest is available here: https://ec.europa.eu/docsroom/documents/37824 (last access: 22 October 2022).

3 In particular, Hidalgo & Hausmann (2009) introduced an empirical methodology to infer the productive capabilities and know-how a country is endowed with by recursively combining information on a country’s diversification (the number of products a country exports with a revealed comparative advantage) and product ubiquity (the number of countries reporting a revealed comparative advantage in that specific product). More complex countries will be those exhibiting a more diversified productive structure (i.e., they report a more diversified export basket of products) signaling that they are equipped with a larger number of capabilities, which in turn allows them to produce less ubiquitous goods and services. Consistently, Hausmann & Hidalgo (2011) found a systematic negative relationship between the diversification of a country’s exports and the ubiquity of its products, meaning that more diversified countries tend to export less ubiquitous products.

4 The importance of production networks as a key feature of the changing organisation of global innovation-based competition dates way back in the literature and has received a timely and careful attention also in this Journal. See Vaccà (1986), Rullani (1989), Vaccà and Zanfei (1989) for an early consideration of the topic, and Castellani et al. (2017) for a more recent reconsideration.
